# Upregulation of miRNA-4776 in Influenza Virus Infected Bronchial Epithelial Cells Is Associated with Downregulation of NFKBIB and Increased Viral Survival

**DOI:** 10.3390/v9050094

**Published:** 2017-04-27

**Authors:** Sreekumar Othumpangat, Nicole B. Bryan, Donald H. Beezhold, John D. Noti

**Affiliations:** 1 Allergy and Clinical Immunology Branch, Health Effects Laboratory Division, National Institute for Occupational Safety and Health, Centers for Disease Control and Prevention, Morgantown, WV 26505, USA; zec1@cdc.gov (D.H.B.); ivr2@cdc.gov (J.D.N.); 2 School of Medicine, West Virginia University, Morgantown, WV 26506, USA; nbryan2@hsc.wvu.edu

**Keywords:** NFKBIB, influenza virus, bronchial epithelial cells, NF-κB, virus survival

## Abstract

Influenza A virus (IAV) infection remains a significant cause of morbidity and mortality worldwide. One key transcription factor that is activated upon IAV infection is nuclear factor Kappa B (NF-κB). NF-κB regulation involves the inhibitor proteins NF-κB inhibitor beta (NFKBIB), (also known as IκB β), which form complexes with NF-κB to sequester it in the cytoplasm. In this study, microarray data showed differential expression of several microRNAs (miRNAs) on exposure to IAV. Target scan analysis revealed that miR-4776, miR-4514 and miR-4742 potentially target NFKBIB messenger RNA (mRNA). Time-course analysis of primary bronchial epithelial cells (HBEpCs) showed that miR-4776 expression is increased within 1 h of infection, followed by its downregulation 4 h post-exposure to IAV. NFKBIB upregulation of miR-4776 correlated with a decrease in NFKBIB expression within 1 h of infection and a subsequent increase in NFKBIB expression 4 h post-infection. In addition, miRNA ago-immunoprecipitation studies and the three prime untranslated region (3’ UTR) luciferase assay confirmed that miR-4776 targets NFKBIB mRNA. Furthermore, uninfected HBEpCs transfected with miR-4776 mimic showed decreased expression of NFKBIB mRNA. Overexpression of NFKBIB protein in IAV infected cells led to lower levels of IAV. Taken together, our data suggest that miRNA-4776 modulates IAV production in infected cells through NFKBIB expression, possibly through the modulation of NF-κB.

## 1. Introduction

Influenza infection is a significant cause of morbidity and mortality causing an estimated 3–5 million infections per year [1]. Upon infection, the virus hijacks the host’s cellular machinery for its survival and replication. One mechanism by which influenza may influence host gene expression is through differential expression of host cell microRNAs (miRNA), which are endogenously produced ~22 nucleotide single-strand RNAs that interact with the three prime untranslated region (3’ UTR) of messenger RNAs (mRNAs) and destabilize the transcripts or degrade them to repress translation [2]. These molecules have been shown to play a key role in the regulation of a diverse array of cellular responses including inflammation and cell death. Since their initial discovery, miRNAs have been developed as both diagnostic tools and therapeutic targets. A number of miRNAs identified are predicted to target key elements of the immune response and cell survival pathways [3].

Nuclear factor Kappa B (NF-κB) plays an important role in regulating several genes that are necessary for cell proliferation, apoptosis and cell survival [4]. NF-κB also activates pro-inflammatory genes in cells at the sites of inflammation in several diseases including infectious diseases [5,6]. In addition, NF-κB itself is activated upon influenza infection [7,8,9]. It was previously believed that activation of NF-κB served as a cellular defense mechanism upon influenza viral infection with production of inflammatory cytokines, including interferon β (IFNβ) [7,10]. Studies conducted in A549 and U1752 cells showed that low NF-κB activity were resistant to influenza A virus (IAV) infection, but cells became susceptible to IAV upon activation of NF-κB. One suggested role of NF-κB signaling is in the endocytosis pathway that is necessary for efficient influenza virus infection (9). Some of the viral proteins are involved in the activation of NF-κB [11]. The viral non-structural protein NS1 acts as a suppressor of NF-κB activation [12]. However, the antagonistic activity of NS1 is not exhaustive for complete blocking of NF-κB [13]. Moreover, other viral components such as hemagglutinin [14], nucleoprotein and matrix proteins can induce NF-κB activation [11]. In vitro studies showed that the NF-κB specific inhibitor, SC75741, expressed in bronchial epithelial cells decreases the propagation of IAV [15]. This effect was subsequently confirmed in an in vivo mouse model in which SC75741 was able to effectively protect mice from the deadly effects of three highly pathogenic influenza strains. Based on these studies, it is hypothesized that IAV has evolved a strategy to activate NF-κB to prevent apoptosis of the host cell and facilitate viral replication.

Regulation of NF-κB is a complex process involving both internal and external cellular stimuli [16]. NF-κB regulation involves a family of inhibitor proteins, NF-kappa B inhibitor beta protein (NFKBIB, also known as IκBβ) that forms a complex with NF-κB and sequesters it into the cytoplasm [17]. This effectively prevents nuclear translocation of NF-κB and subsequent transcription of its target genes [18]. Phosphorylation of the serine residues on NFKBIB proteins directs them for degradation and releases NF-κB from the complex resulting in its translocation to the nucleus [19]. Given that NF-κB plays a role in influenza infection, our aim was to determine the role of specific miRNAs in regulating the NF-κB pathway. In gastric cancer cell lines, miR-20a was shown to target NFKBIB [20], and miR-182-5p was shown to target NFKBIB in breast cancer cells [21]. However, possibly due to tissue specific expression, we saw no significant change in these miRNAs in IAV infected alveolar lung epithelial (A549) cells. In our miRNA microarray analysis, we show that miR-4514, miR-4742, and miR-4776 are significantly upregulated in IAV infected A549 cells and target scan [22] analysis showed that these miRNAs may target NFKBIB mRNA. We show that these three miRNAs are differentially expressed in IAV infected human primary bronchial epithelial cells (HBEpCs) and that miR-4776 specifically downregulates expression of NFKBIB that leads to activation of NF-κB and increased survival of IAV in HBEpCs cells.

## 2. Materials and Methods

### 2.1. Cell Culture

HBEpCs were purchased from PromoCell GmbH (Heidelberg, Germany) and sub-cultured in media and growth factors recommended by the supplier. A549 cells (CCL-34, American Type Culture Collection (ATCC), Manassas, VA, USA) were cultured in standard F12K medium with heat inactivated 10% fetal bovine serum (FBS), 100 IU/mL penicillin and 100 µg/mL streptomycin sulfate. Madin–Darby canine kidney (MDCK) cells were used for the propagation of influenza virus. MDCK cells were cultured in Minimum Essential Medium (MEM) (ATCC) supplemented with 10% FBS, 100 IU/mL penicillin and 100 µg/mL streptomycin sulfate [22].

### 2.2. Viruses and Their Infections

Influenza virus A/WSN/33 (H1N1) was a kind gift from Prof. Robert A. Lamb (Northwestern University, Chicago, IL, USA) and cultivation and maintenance of the virus was carried out as described earlier [23].

All infections of airway epithelial cells were performed in six-well plates at a dose of 1.0 multiplicity of infection (MOI) unless otherwise specified. Controls referred are the cells that were mock infected. Six-well plates were seeded with 5 × 10^5^ cells per well and grown to 80% confluence. Cells were rinsed with phosphate buffered saline (PBS), and then the virus diluted in modified Hank’s Balanced Salt Solution (HBSS) was added to each well. After 45 min incubation at 37 °C, excess virus was washed off with PBS. Fresh F12 media was added containing 1 µg/mL of tosyl phenylalanyl chloromethyl ketone (TPCK)-trypsin (Sigma-Aldrich, St Louis, MO, USA) and incubated at 37 °C and 5% CO_2_. TPCK was not added to A/WSN/33 virus. Cells were harvested at different time intervals and used for protein and RNA studies.

### 2.3. Real-Time Reverse Transcription Polymerase Chain Reaction

After the experimental treatment of the HBEpCs and A549 cells total RNA was extracted from these cells with the RNeasy plus Mini Kit (Qiagen, Germantown, MD, USA). RNA was quantified with a NanoDrop spectrophotometer (Thermofisher Scientific, Foster City, CA, USA). Total RNA was then reverse transcribed with the High-Capacity complementary DNA (cDNA) Reverse Transcription Kit (Applied Biosystems, Foster City, CA, USA). All reverse transcription-polymerase chain reactions (RT-PCRs) were carried out using standard TaqMan primers for NFKBIB (assay id # Hs00182115_m1), NF-κB (assay ID# Hs00765730_m1), and glyceraldehyde phosphate dehydrogenase (GAPDH) (assay ID # Hs03929097_g1), which were purchased from Applied Biosystems (Thermofisher Scientific). Fold change in expression was determined using the ∆∆ct method after normalizing to GAPDH [22].

miRNA was isolated using the miReasy Kit (Qiagen) and analyzed by RT-PCR with the TaqMan MicroRNA Reverse Transcription Kit (Lifetechnologies, Foster City, CA, USA) and specific primers for miR-4776 (assay # 462695), 4514 (assay # 462737), 4742 (assay # 463053) and the U6 (assay # 001973) control. Influenza matrix gene expression was quantified and reported as influenza copy number. RT-PCR was performed using TaqMan assay with matrix-specific primers, as reported earlier [24]. The M segment of the RT-PCR was specific for viral RNA. A standard curve was generated from the cloned influenza IAV matrix gene by RT-PCR for IAV quantification.

### 2.4. miRNA Microarray

A549 cells were infected with IAV for 3 h and the RNA was extracted using the Exiqon miRCURY locked nucleic acid (LNA) miRNA extraction kit (Exiqon, Vedbaek, Denmark). The quality of the total RNA was verified in an Agilent 2100 Bioanalyzer profile (Agilent Technologies, Inc., Santa Clara, CA, USA). In addition, 750 ng total RNA from both sample and reference was labeled with Hy3 ™ and Hy5 ™ fluorescent label, respectively, using the miRCURY LNA ™ microRNA Hi-Power Labeling Kit, Hy3 ™/Hy5 ™ (Exiqon) according to the procedure described by the manufacturer. The Hy3 ™-labeled samples and a Hy5 ™-labeled reference RNA sample were mixed pairwise and hybridized to the miRCURY LNA ™ microRNA Array 7th Gen (Exiqon), which contains capture probes targeting all miRNAs for human, mouse or rat registered in the miRBASE 18.0. The hybridization was performed according to the miRCURY LNA ™ microRNA Array Instruction manual using a Tecan HS4800 ™ hybridization station (Tecan, Grodig, Austria). After hybridization, the microarray slides were scanned and stored in an ozone free environment to prevent potential bleaching of the fluorescent dyes. The miRCURY LNA ™ microRNA array slides were scanned using the Agilent G2565BA Microarray Scanner System (Agilent Technologies) and the image analysis was carried out using the ImaGene ® 9 (miRCURY LNA ™ microRNA Array Analysis Software, Exiqon). The quantified signals were background corrected and normalized using the global Lowess (LOcally WEighted Scatterplot Smoothing) regression algorithm. The data obtained were subjected to statistical analysis and differentially regulated miRNAs in infected and uninfected cells were identified and reported by Exiqon.

### 2.5. Transfection Studies

HBEpCs were transfected with a miRNA-4776 or 4514 inhibitor oligonucleotide (complementary strand to miRNAs) or a miRNA-4776 or 4514 mimic oligonucleotide (corresponding to the miRNA-sequence) (Life Technologies, Carlsbad, CA, USA) using the lipid-based Lipofectamine 2000 reagent diluted in Opti-MEM-I reduced serum medium (Life Technologies) according to the protocol provided by the supplier. Briefly, HBEpCs were grown to 80% confluence in six-well plates. Transfection complexes were directly applied to the cells (final concentration of 50 nM) and the plates were incubated in a humidified chamber with 5% CO_2_ at 37 °C. After 6 h of transfection, the medium was replaced with fresh complete medium. As negative control, cells were transfected with a scrambled oligonucleotide (Life Technologies). To evaluate the effects of miRNA-4514 and 4776 in the context of viral infection, cells after 48 h of transfection were infected with IAV at a MOI of 1. Virus were allowed to attach to the cells for 45 min at 37 °C, then the excess virus was washed off and fresh medium containing 1 µg/mL of TPCK trypsin (Sigma-Aldrich) was added. Cells were then incubated for another 4 h. Following the incubation, cells were harvested at different time intervals and used for protein and RNA studies.

### 2.6. Imaging with Confocal Microscopy

HBEpCs cells were grown on chamber slides overnight (Chamber slide ™, Lab-TekII, Thermo Fisher Scientific, Rochester, NY, USA) to 80–90% confluence. Cells were then exposed to IAV for 4 h. Subsequently, cells were washed with PBS and fixed with 4% methanol-free formaldehyde (Polysciences Inc., Warrington, PA, USA). Immunofluorescent staining was done as described earlier [23] and stained with an antibody that recognizes the phosphorylated form of NF-κBp65, rabbit anti-phospho-NF-κB p65 antibody (Millipore, Billerica, MA, USA) for 1 h, followed by Alexa-488 conjugated anti-rabbit secondary antibody (Life Technologies). HBEpCs overexpressing NFKBIB were infected with IAV and then stained with NFKBIB antibody (Cell signaling, Danvers, MA, USA) and NS1 antibody (Invitrogen, Foster City, CA, USA), followed by appropriate secondary antibodies (Alexa 488 and Alexa-555). The glass slides were mounted with 4′,6-diamidino-2-phenylindole (DAPI)-Prolong Gold anti-fade reagent (Life Technologies) and protected with cover slips. Photomicrographs were made using a Zeiss Laser Scanning Microscopy (LSM)-510 (Carl Zeiss AG, Obertochen, Germany) confocal microscope.

### 2.7. Overexpression of NFKBIB

HBEpCs cells were transiently transfected with the open reading frame (ORF) of NFKBIB cloned in pCMV6-Entry vector from Origene (Origene, Rockville, MD, USA) and lipofectamine 2000 (Invitrogen). Cells transfected with an empty pCMV6 vector were used as control. Following transfection for 48 h, cells were infected with IAV at 1 MOI for another 10 h. Cells were harvested at different time points as described in Results and Discussion. RNA was isolated and RT-PCR for the viral matrix copy number as well as the NFKBIB transcripts were done as described earlier.

#### 2.7.1. Validation of miRNA by Ago Immunoprecipitation

Immunoprecipitation (IP) of the miRNA was done according to the instructions from the kit available from Active Motif (Active Motif, Carlsbad, CA, USA). Briefly, HBEpCs were grown to 80% confluency in six-well plates and were transfected with 25 nM mimics of miR-4776 or miR-4514 or a negative control (scrambled oligonucleotide of mimic or inhibitor) for 24 h. An equal number of cells were taken for the IP to minimize variability. IP uses G-coupled magnetic beads and pan-Ago antibody that recognizes Ago 1, Ago 2 and Ago 3 to precipitate the miRNA/mRNA complex. An isotype antibody control was also run in parallel. IP was done as described in the manufacturer’s protocol. The precipitated complex was collected and the RNA purified from the complex using trizol reagent. The RNA was converted to cDNA with the High Capacity cDNA Reverse Transfection Kit (Applied Biosystems) and specific primers for NFKBIB were used for RT-PCR. The data was analyzed by comparing the cells transfected with mimic miRNA or negative control oligonucleotide and the fold enrichment of NFKBIB was calculated from the Ago and isotype antibody preparations as described by the manufacturer.

#### 2.7.2. Luciferase Assay

The 3’ UTR sequence of NFKBIB was cloned downstream of the firefly luciferase gene. The transcript level is regulated by its interaction with miRNAs resulting in reduced luciferase activity that is measured by the Promega Dual Luciferase Assay (Madison, WI, USA). NFKBIB 3’ UTR reporter plasmid was synthesized by Origene (Origene). HBEpCs cells were transfected on a 96-well plate with a complex containing transfection agent (Lipofectamine 2000, Life Technologies), NFKBIB UTR reporter plasmid, and 25 nM of either the miR-4776 mimic or negative control (a scrambled oligonucleotide that is not a target of any gene). After 18 h of transfection, the medium was replaced with complete medium and the cells were incubated for an additional 16–18 h. Cells were then collected and lysed in lysis buffer supplied by the manufacturer. Luciferase activity was assessed in microtiter plates using the Promega Dual Luciferase Reporter Assay system according to the manufacturer’s instructions (Dual-Glo ® Luciferase Assay, Promega). The plates were read on a Glomax ® 96-well microplate Luminometer (Promega). The relative Renilla luciferase (Promega) activity was calculated by normalizing transfection efficiency to the firefly luciferase activity.

### 2.8. Western Immunoblotting

HBEpCs were transfected with miR-4776 mimic and then mock infected or infected at a MOI of 1 IAV. Cells were also transfected with a negative control of mimic. Four hours post infection, the cells were lysed using a cytoplasmic protein extraction buffer containing protease inhibitors (Thermofisher Scientific, Foster City, CA, USA). The isolated proteins were electrophoresed on a 10% SDS-polyacrylamide gel (sodium dodecyl sulfate-polyacrylamide gel electrophoresis (SDS-PAGE), Pre-cast gel, BioRad (Hercules, CA, USA). Separated proteins were transferred to a nitrocellulose membrane (Millipore) and the membrane was blocked using Odyssey Blocking Buffer (LI-COR Biosciences, Lincoln, NE, USA). Western blot analysis was carried out using mouse monoclonal anti-phospho-NF-κB p65 (Millipore) and mouse monoclonal anti-GAPDH (Abcam, Cambridge, MA, USA) and NS1 of IAV (Invitrogen). Appropriate mouse and rabbit IR Dye 680 or 800 secondary antibodies (LI-COR Biosciences, Lincoln, NE, USA) were used. Near-infrared fluorescence detection was performed on the Odyssey Imaging System (LI-COR Biosciences).

### 2.9. Viral Plaque Assay

MDCK cells were cultured in six-well tissue culture plate and incubated at 35 °C in a humidified CO_2_ incubator. Confluent monolayers of the cells were washed with PBS and inoculated with 800 μL of serially diluted cell culture supernatant collected from the HBEpC cells exposed to IAV and incubated for 45 min at 35 °C. Immediately after incubation, the cells were washed twice with 2 mL of PBS, overlaid with supplemented Dulbecco’s Modified Eagle’s medium (DMEM)/F12 containing 0.6% agarose (Oxoid Ltd., Basingstoke , Hampshire, UK) and incubated at 35 °C for another 60 h. Cells were then fixed with 10% formalin and the agarose removed with tap water and stained with 1.0% crystal violet and plaque forming units (PFU) were counted.

### 2.10. Statistical Analysis

One-way analysis of variance (ANOVA) was used to analyze RT-PCR data and post-hoc pairwise multiple comparisons between means were performed using the Holm–Sidak method with a *p*-value of <0.05 considered statistically significant using Sigma stat version 11.0 for Windows (Systat Software, Chicago, IL, USA).

## 3. Results and Discussion

### 3.1. Microarray Analysis

**Figure 1:**
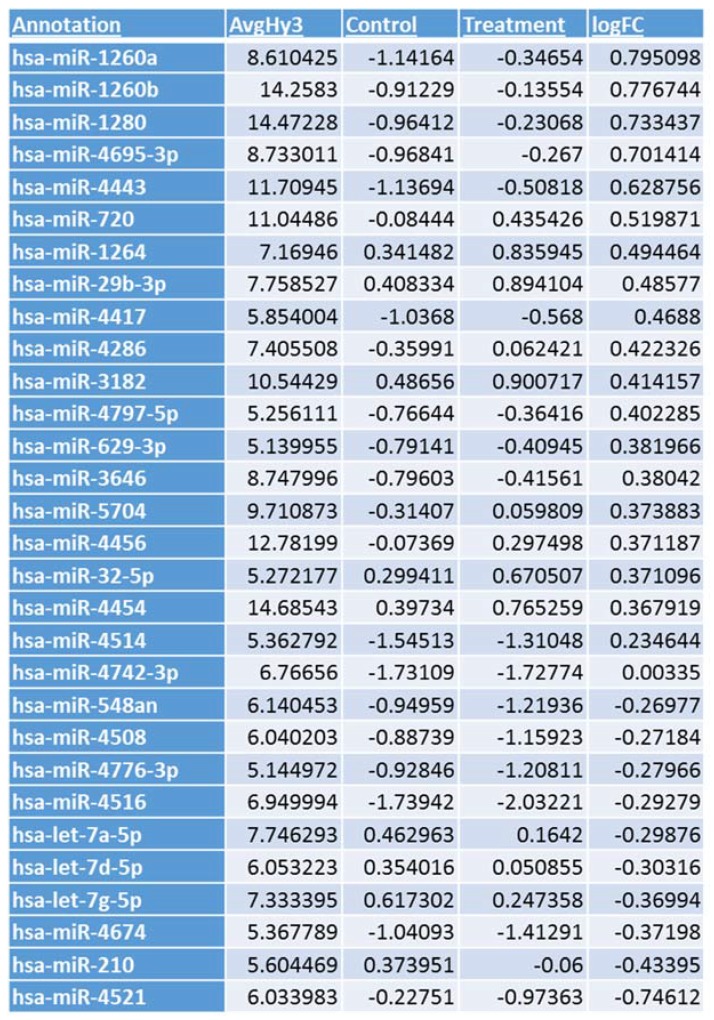
Influenza A virus (IAV) infection induced differential expression of microRNAs (miRNAs). Microarray analysis for miRNA expression was performed with RNA extracted from IAV infected A549 cells for 3 h. The log fold changes in expression of miRNAs that are up or downregulated on exposure to IAV are shown from the microarray. AvgHy3-Average signal intensity, logFC—log fold change, Control-Mock; Treatment-Infected with H1N1.

**Figure 2:**
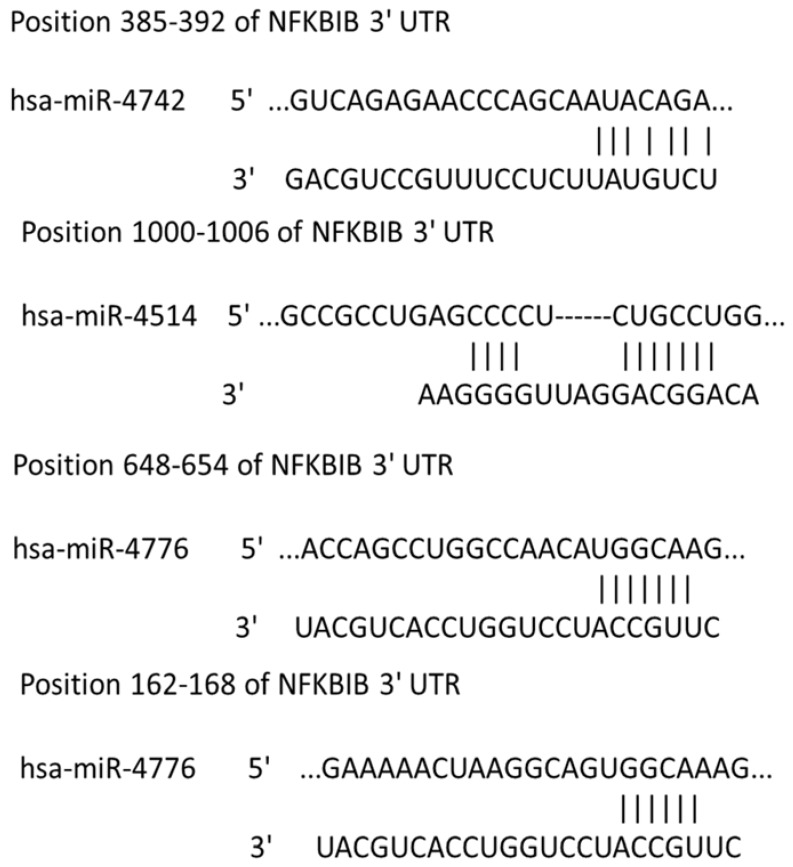
Target scan analysis of nuclear factor Kappa B inhibitor beta (NFKBIB) three prime untranslated region (3’ UTR). A search of the sequences for miRNAs miR-4514, 4742, and 4776 showed putative target binding sites in NFKBIB messenger RNA (mRNA).

The virus host paradigm provides a mechanism by which RNA viruses can regulate several host genes through the modulation of host-specific miRNAs [25,26,27]. To identify influenza-induced changes in host miRNA expression, a microarray analysis of host miRNAs differentially regulated following IAV infection was performed. A549 cells exposed to IAV showed differential expression of miRNAs as shown in Figure 1. We focused on the regulatory mechanisms for NF-κB expression, as it may have a role in the replication and survival of IAV [9,10]. Little is known regarding miRNAs that regulate the expression of NF-κB negative regulator, NFKBIB, and recent studies indicate that miR-20a targets NFKBIB in gastric cancer cells [20]. However, miR-20a did not show any significant differential expression in A549 cells infected with IAV. In contrast, miR-4514 exhibited a slight upregulation and miR-4776 was downregulated in A549 cells infected with IAV (Figure 1) and target scan analysis revealed that miR-4514 and miR-4776 are putative targets of NFKBIB (Figure 2). We added miR-4742-3p to our validation study since it showed significant upregulation at 18 h exposure to IAV (data not shown), and target scan predicted it as a target of NFKBIB. miRNA-4776 was of particular interest in that two putative binding sites for this miRNA are present in the NFKBIB 3’ UTR region, one at position 162–168 and another at position 648–654. Even though several microarray studies have shown a wide range of miRNAs being modulated by IAV infection, very few miRNAs were studied in detail to understand the target and role in IAV replications. Moreover, the induction of miRNA also depends on the cell type, incubation time and the MOI of virus used. Previous studies also have shown that many target genes of miRNAs contain several miRNA binding sites and the level of translational repression exponentially increases with the number of miRNA binding sites in the 3’ UTR region [28]. Therefore, this potential increase in binding of miRNA-4776 to the 3’ UTR of NFKBIB may lead to reduced expression of this repressor and trigger increased activity of NF-κB. We further examined the role and specificity of these miRNAs in NFKBIB expression (Table S1 shows the complete microarray data).

### 3.2. miRNA and NFKBIB Expression

**Figure 3:**
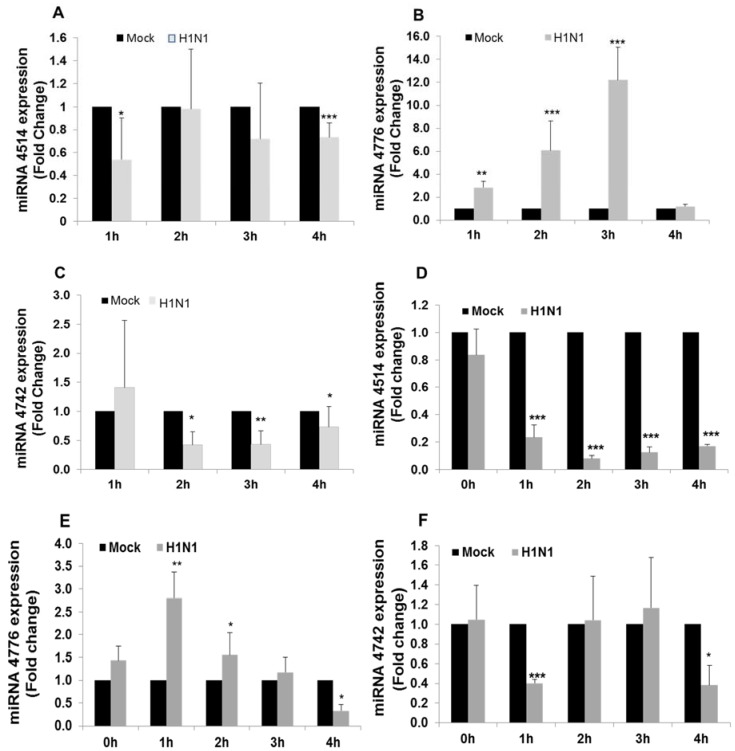
miRNA expression profile on exposure to IAV compared with mock and infected cells. (**A**) expression pattern of miR-4514. A549 cells were infected with IAV at multiplicity of infection (MOI) of 1 for 4 h and samples were taken every hour, miRNAs extracted and analyzed by reverse transcription-polymerase chain reaction (RT-PCR). *** *p* < 0.001; (**B**) expression pattern of miR-4776. A549 cells were infected with IAV at MOI of 1 for 4 h and samples were taken every hour, miRNAs extracted and analyzed by RT-PCR. ** *p* < 0.01, *** *p* < 0.001; (**C**) expression pattern of miR-4742. A549 cells were infected with IAV at MOI of 1 for 4 h and samples were taken every hour, miRNAs extracted and analyzed by RT-PCR. Data is from three independent experiments. * *p* < 0.05, ** *p* < 0.01; (**D**) expression pattern of miR-4514. Human bronchial epithelial cells (HBEpCs) infected with IAV at MOI 1 for 4 h and samples were taken every hour, miRNAs extracted and analyzed by RT-PCR. Data is from three independent experiments. *** *p* < 0.001; (**E**) expression pattern of miR-4776. HBEpCs were infected with IAV at MOI of 1 for 4 h and samples were taken every hour, miRNAs extracted and analyzed by RT-PCR. Data is from three independent experiments. * *p* < 0.05, ** *p* < 0.01; and (**F**) expression pattern of miR-4742. HBEpCs were infected with IAV at MOI of 1 for 4 h and samples were taken every hour, miRNAs extracted and analyzed by RT-PCR. Data is from three independent experiments. * *p* < 0.05, *** *p* < 0.001. Expression of each miRNA was normalized to expression of U6 miRNA.

Our earlier studies [23] showed that the host cellular response (for cell survival) in response to the viral invasion occurs at the early stages of infection (up to 3 h). Thus, it is appropriate to look for changes that occur at early hours of infection, before the virus take control of the cell metabolism and signaling. To validate the initial microarray analysis, A549 cells were infected with IAV for 1 to 4 h and miRNA expression was analyzed by RT-PCR. MiR-4514 expression was significantly decreased at 1 h exposure to IAV compared to mock infected control (Figure 3A), increased at 2 h to the level observed at 3 h in the microarray analysis, but then decreased again at 3 h and 4 h. The data from RT-PCR is more sensitive and reliable than microarray analysis and may account for the apparent discrepancy in expression between these assays at 2–3 h. Expression of miR-4776 in infected cells increased significantly throughout the (1–3 h) infection, and expression peaked (12 fold increase) by 3 h and then sharply decreased by 4 h (Figure 3B), a similar trend was seen by array analysis with the difference in incubation time of 1 h, the difference in time again could be due to the increased viral copies in array. The array data showed a decrease at 3 h, and the difference in time with validation and array could be attributed to the difference in MOI. Expression of miR-4742 decreased in the initial 3 h of infection and then increased by 4 h to that level in the mock infected control (Figure 3C). This return to basal level of miR-4742 after 4 h of infection was also seen after 3 h of infection in the initial microarray analysis, which may be due to the higher MOI of 3 used in the microarray analysis. The high MOI used in the array analysis was to ensure that every cell in culture was infected with one or more virus, whereas in the validation studies, we reduced the MOI to 1, so that the overburden of the virus on the culture cells could be avoided and the host cells response to viral infection could be measured. A549 cells are transformed lung epithelial cells that could also produce variation in expression compared to primary normal lung cells, hence we used human primary bronchial epithelial cells to confirm the repeatability of the results.

HBEpCs cells were infected with IAV at MOI of 1 for 1–4 h and miRNA expression was analyzed by RT-PCR. MiR-4514 expression was significantly decreased throughout the 4 h exposure compared to mock infected control (Figure 3D). Expression of miR-4776 showed a significant increase at 1 h of IAV infection (2.8 fold) but then gradually decreased to that of the mock control after 3 h and decreased further by 4 h (Figure 3E). Expression of miR-4742 initially decreased after 1 h of infection and then returned to that of the mock control after 2 h and 3 h, followed again by a sharp reduction in expression after 4 h (Figure 3F). The patterns of expression of the three miRNAs in HBEpCs was similar (but not exactly) as was found in A549 infected cells with expression of miR-4776 showing the most consistency, although expression of this miRNA peaked earlier in the infection in HBEpCs.

**Figure 4:**
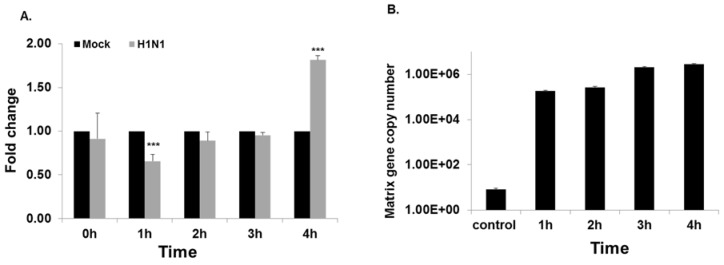
Influenza infection modulates NFKBIB expression: (**A**) HBEpCs grown to 80% confluency were infected with IAV at an MOI of 1 or mock for 4 h and samples were taken every hour. RNA was extracted and complementary DNA (cDNA) was synthesized, and used for RT-PCR analysis. NFKBIB expression was analyzed and normalized to glyceraldehyde phosphate dehydrogenase (GAPDH), and data are expressed as ± standard error of the mean (SEM), *** *p* < 0.001, *n* = 3 independent experiments; (**B**) influenza virus matrix gene copy number was analyzed from the RNA extracted from HBEpCs infected with IAV for 4 h; *n* = 3 independent experiments analyzed in duplicates. Control is the mock infected.

We compared the expression levels of NFKBIB mRNA with those of the three miRNAs in HBEpC cells. Expression of NFKBIB mRNA significantly decreased 1.6-fold after 1 h of infection, returned to the level of the mock infected control after 2 h and 3 h, and then significantly increased ( 2-fold) after 4 h exposure to IAV (Figure 4A). When the expression of the three miRNAs (miR-4415, 4742, 4776) was compared with that of NFKBIB mRNA, only miRNA-4776 showed an inverse correlation, as would be expected if NFKBIB mRNA expression is regulated by the specific miRNA. The influenza copy number gradually increased after 4 h of exposure to IAV (Figure 4B). A classical approach to identify the actual target mRNA of these miRNA is by introducing mimics and inhibitors of the specific miRNA and evaluating the target gene expression [29,30]. We further examined the role and specificity of miR-4776 and miR-4514 miRNAs to NFKBIB but did not study miR-4742-3p, as it did not appear to have a pattern of expression, suggesting it is involved in NFKBIB expression.

### 3.3. Functional Analysis of miR-4776 and miR-4514

**Figure 5:**
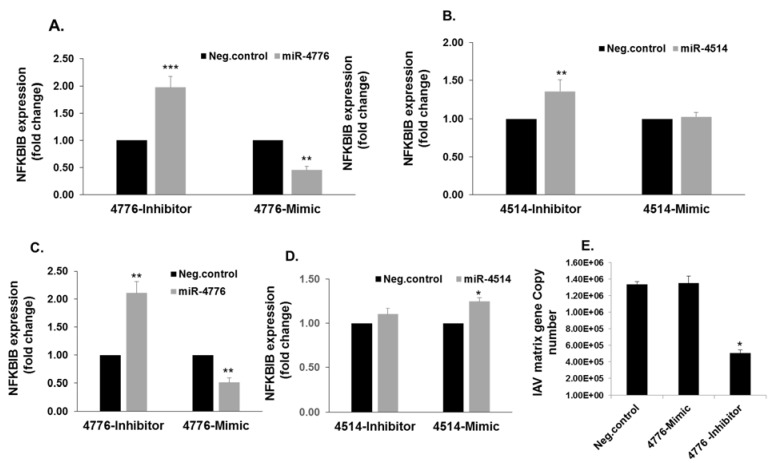
Downregulation of miR-4776 induce NFKBIB expression: (**A**) HBEpCs were transfected with miR-4776 inhibitor or mimic or negative control. After 48 h of incubation, RNA was extracted and converted to cDNA and used for RT-PCR. GAPDH was used as the housekeeping gene. Data are expressed as ± SEM, *** *p* < 0.001, ** *p* < 0.01 (*n* = 3); (**B**) HBEpCs were transfected with miR-4514 inhibitor or mimic or the negative control. After 48 h of incubation, RNA was extracted and converted to cDNA and used for RT-PCR. The relative abundance of NFKBIB was measured in cells and the data normalized to the abundance of GAPDH. Data are expressed as ± SEM, * *p* < 0.05, ** *p* < 0.01 (*n* = 3); (**C**) HBEpCs were transfected with miR-4776 mimic or inhibitor or a negative control. After 48 h of incubation, the transfected cells were either mock infected or infected with 1 MOI IAV for an additional 4 h. Relative expression of NFKBIB was analyzed by RT-PCR. Data are expressed as ± SEM, ** *p* < 0.01 compared the cells transfected with the negative control, (*n* = 3); (**D**) HBEpCs transfected with miR-4514 mimic or inhibitor or a negative control. After 48 h of incubation, the cells were infected with 1 MOI of IAV for another 4 h. After 4 h of incubation, RNA was extracted and converted to cDNA and used for RT-PCR. The relative NFKBIB abundance was measured in infected cells, and GAPDH was used as the internal control. Data are expressed as ± SEM, * *p* < 0.05 compared the cells transfected with the negative control, (*n* = 3); (**E**) abundance of NFKBIB modulates viral replication. HBEpCs were transfected with miR-4776 mimic or inhibitor or a negative control. Following infection, the transfected cells were infected with 1 MOI of IAV for an additional 4 h. After 4 h of incubation, RNA was extracted and converted to cDNA and used for RT-PCR. Matrix copy number in cells transfected with either miR-4776 mimic or inhibitor or negative control is shown. Data are expressed as ± SEM, ** *p* < 0.001 (*n* = 4).

To examine the functionality of these miRNAs, HBEpCs were transfected with the mimic or inhibitors of miR-4514 and miR-4776 and the changes in NFKBIB mRNA expression were determined. The miR-4776 inhibitor increased the NFKBIB mRNA expression 1.75-fold and the exposure to miR-4776 mimic decreased the NFKBIB mRNA expression approximately 50% in uninfected cells transfected with the negative control (Figure 5A). In contrast, the change in NFKBIB expression in the presence of miR-4514 mimic was not consistent with a role in regulating NFKBIB mRNA (Figure 5B). HBEpCs transfected with miR-4776 inhibitor and subsequently infected with IAV showed an increase in NFKBIB expression, and a decrease with mimic (Figure 5C), whereas miR-4514 mimic increased NFKBIB and inhibitor did not significantly alter NFKBIB expression in IAV infected cells (Figure 5D). This apparent lack of expected inverse correlation between miR-4514 expression and NFKBIB expression may be due to an indirect effect on the NFKBIB pathway, as miRNA target multiple mRNAs.

The increased expression of NFKBIB resulting from transfection of miR-4776 inhibitor in HBEpCs also is associated with a significant decrease in IAV matrix gene copy number as evidenced in the 65% decrease in copy number (Figure 5E). This result is consistent with the concept that IAV is dependent on the NF-κB pathway [10] and the increase in NFKBIB reduced the level of active NF-κB available for inducing the transcription of viral genes. In contrast, further decreasing the level of NFKBIB mRNA with miR-4776 mimic did not alter the matrix copy number in infected cells, suggesting that there was a sufficient number of NF-κB molecules to support IAV replication (Figure 5E). miRNAs are known to induce changes in the cellular physiology by different mechanisms, and each miRNA has multiple gene targets [29,31,32]. The exogenous administration of miRNA inhibitor to antagonize influenza replication in airway epithelial cells could be a potential therapeutic strategy to explore for controlling influenza infections.

**Figure 6:**
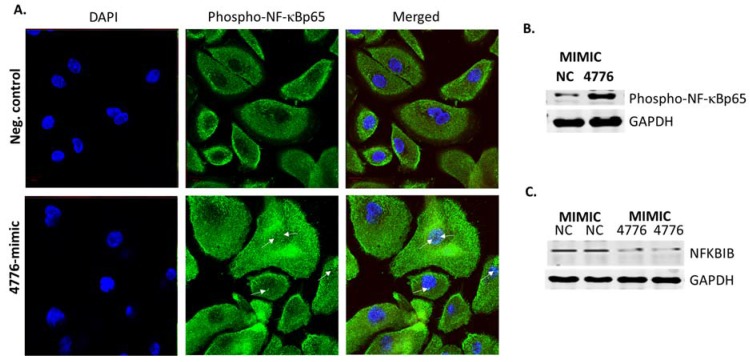
Immunofluorescence of IAV infected cells showing the expression of NF-κB. (**A**) HBEpCs were transfected with miR-4776 mimic or a negative control. After 48 h of incubation, the cells were infected with 1 MOI of IAV for another 4 h. (NC represents the negative control and is a scrambled oligonucleotide) (top panel), miRNA-4776 mimic (bottom panel). The expression of activated phospho-NF-κB p65 protein (green) was determined by immunofluorescence. 4′,6-diamidino-2-phenylindole (DAPI) (blue) stained the nucleus of the cells. Arrows indicate the NF-kB translocated into the nucleus; (**B**) HBEpCs transfected with miR-4776 mimic or a negative control. After 48 h of incubation, the cells were infected with 1 MOI of IAV for another 4 h. Western blot analysis of cells transfected with mimic miR-4776 showing increased phospho-NF-κB p65 protein; (**C**) immunoblot showing the decreased expression of NFKBIB protein in cells transfected with miR-4776 and infected with IAV. HBEpCs transfected with miR-4776 mimic or a negative control. After 48 h of incubation, the cells were infected with 1 MOI of IAV for another 4 h. GAPDH is used as the housekeeping protein.

Unstimulated cells contain a pool of NF-κB dimers in the cytosol that are stoichiometrically bound with the inhibitors [16]. NFKBIB is complexed with NF-κB but upon infection or external stimuli activated NF-κB is released [17], and subsequently translocated to the nucleus where it induces transcription of genes. HBEpCs transfected with mimic miR-4776 followed with IAV infection for 4 h were monitored for the expression of NF-κB by confocal microscopy (Figure 6A) and compared to the negative control. The cells were stained with an antibody to phosphorylated NF-κBp65 that specifically binds to the p65 component of active NF-κB (green), and the nucleus was stained with DAPI (blue). Active phosphorylated NF-κB was significantly higher in miR-4776 transfected cells compared to the negative control mimic transfected cells infected with IAV (Figure 6B). This data suggests that mimic miR-4776 lowers the levels of NFKBIB mRNA and the amount of NFKBIB protein available to bind with NF-κB results in an increased level of active NF-κB. This is consistent with previous studies that showed NFKBIB, once released from the NF-κB/NFKBIB complex, undergoes degradation and leads to the phosphorylation and activation of NF-κB [18,33]. We further confirmed the results obtained by confocal microscopy with Western blot analysis. Data presented in Figure 6B shows that phosho-NF-κB p65 is overexpressed in cells transfected with the miR-4776 mimic compared to the negative control mimic transfected cells. The 4 h time point of infection was selected based on the results (Figure 3E and Figure 4A) wherein 4 h exposure to IAV showed a reduction in miR-4776 expression and upregulation of NFKBIB transcripts. Moreover, the miR-4776 mimic transfected cells showed a reduction in NFKBIB protein compared to the control cells transfected with negative control (Figure 6C). Taken together, the results from this study clearly indicates that miR-4776 has a significant role in regulating NFKBIB expression to alter the level of active NF-κB.

**Figure 7:**
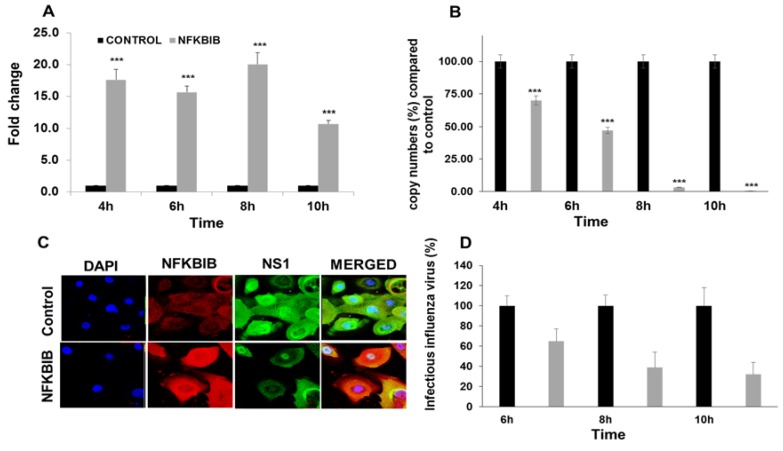
NFKBIB overexpression leads to reduction in IAV M gene copy number. (**A**) HBEpCs grown to 80% confluency were transfected with NFKBIB plasmid or control plasmid for 36–48 h. NFKBIB overexpressing HBEpCs were infected with 1 MOI of IAV for another 10 h, and the expression of NFKBIB was analyzed using RT-PCR. *** *p* < 0.001 (*n* = 3); (**B**) NFKBIB overexpressing HBEpCs were infected with 1 MOI of IAV for another for 10 h, and the IAV matrix gene copy numbers were analyzed using the matrix gene primers, and a standard curve for the matrix gene. *** *p* < 0.001(Black—Control plasmid, Grey—NFKBIB gene overexpressing); (**C**) HBEpCs overexpressing NFKBIB gene were cultured on slides and infected with IAV of 1 MOI. Cells were then fixed and stained with NFKBIB (red) and NS1 (influenza) antibodies (green), followed by respective fluorescent secondary antibodies. Nucleus (blue) was stained with DAPI. Slides were observed under the Laser Scanning Microscopy (LSM)-510 confocal microscope using an oil 63X objective; (**D**) HBEpCs grown to 80% confluency were transfected with NFKBIB plasmid or control plasmid for 36–48 h. NFKBIB overexpressing HBEpCs were infected with 1 MOI of IAV for another 10 h. Culture supernatant were collected at different time points. Percent infectious influenza virus was determined by viral plaque assay from culture supernatant. Percent calculations were normalized to control cells infected with IAV.

To validate the role of NFKBIB, we used two classic methods: silencing of NFKBIB by small interfering RNA (siRNA) and overexpression of NFKBIB by plasmid DNA. Four NFKBIB siRNA oligonucleotides were obtained from Dharmacon (GE, Pittsburgh, PA, USA) but were not effective in reducing the expression of NFKBIB mRNA in HBEpCs. All four siRNA oligonucleotides individually or in combination failed to produce a reduction of NFKBIB transcripts (data not shown). Plasmids carrying the ORF of NFKBIB were transiently transfected in HBEpCs. After 24–36 h of transfection, cells were infected with IAV (1 MOI) for up to 10 h. The results (Figure 7A) indicate that there was an increase (11–21 fold) in NFKBIB expression in cells transfected with the ORF of NFKBIB. When HBEpCs were transfected with NFKBIB ORF plasmids, a significant reduction in IAV copy numbers was observed (Figure 7B). Confocal analysis of the cells overexpressing NFKBIB (Red) infected with IAV (Figure 7C) shows reduced expression of influenza NS1 protein (green) demonstrating the low levels of IAV protein in cells. We selected influenza NS1, as it has been shown to have a significant impact on host cell gene expression and a central role in inhibiting interferon and activating NF-κB and PI3-K pathways [34,35]. The results from these studies show that overexpression of NFKBIB blocks NF-κB activity needed for viral replication. The essential role of NF-κB in viral replication has been well documented [6]. The results corroborate the effect observed with the miR-4776 inhibitor, which increased NFKBIB expression and led to a reduction in matrix gene copy numbers. Figure 7D showed a reduction in viable IAV in cells overexpressing NFKBIB protein compared to the control cells infected with IAV

### 3.4. miRNA Target Confirmation Studies

**Figure 8:**
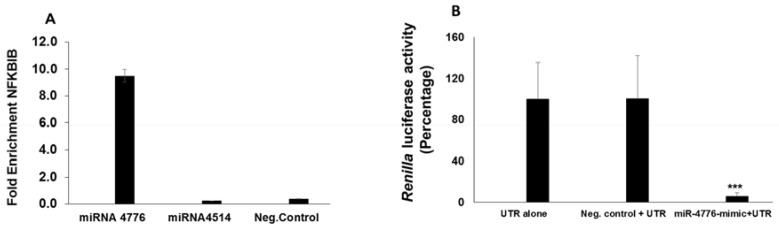
Confirmation of miR-4776 target by Argonaut Immunoprecipitation and luciferase 3’ UTR assay. (**A**) HBEpCs were transfected with miR-4776 mimic, miR-4514 mimic or negative control mimic, and, after 24–36 h. the cells were collected and immunoprecipitated using the Ago or isotype antibody. RNA was isolated and the fold enrichment of NFKBIB transcript was calculated. Independent experiments (*n* = 2) were analyzed in duplicate; (**B**) HBEpCs grown to 80% confluency were co-transfected with the luciferase reporter construct and with either mimic miR-4776 or negative control and after 36 h, cells were infected with IAV at MOI of 1 for 4 h. Luciferase activity was measured using the Dual luciferase assay kit as described by the manufacturer. *** *p* < 0.001, Error bars represent the standard error from three independent experiments.

miRNA is incorporated with Argonaute (Ago) proteins into the RNA-induced silencing complex [36,37], where miRNA guides the complex to partial complementary binding sites located in the 3’ UTR regions [37] of target mRNAs to induce translational repression or degradation of mRNAs. To confirm the specificity of miR-4776 to NFKBIB mRNA, we approached this in two ways, using an Ago antibody pulldown method, and using a 3’ UTR luciferase assay. The Ago protein binds the miRNA to enable the miRNA to base pair with the target mRNA [36], which can be immunoprecipitated (IP) and separated for target identification. Ago was precipitated using a pan-ago antibody (specific for ago-1, ago-2 and ago-3) and protein G magnetic beads (Active Motif kit). HBEpCs were transfected with either miR-4776 or miR-4514 mimic or the negative control mimic. The results presented in Figure 8A show a 10 fold increase in enrichment of NFKBIB mRNA obtained from the IP of miR-4776 mimic transfected cells compared to the negative control mimic transfected cells. Moreover, transfection of miRNA-4514 did not lead to an enrichment of NFKBIB mRNA, suggesting that NFKBIB mRNA binds to miR-4776 and not with miR-4514.

We then addressed the specificity of miR-4776 to target NFKBIB mRNA using the 3’ UTR Luciferase Reporter Assay. Luciferase UTR experiments are considered the confirmatory experiments in miRNA target identification in various studies utilizing miRNA [38,39]. The 3’ UTR of NFKBIB predicted to interact with miR-4776 was cloned into a reporter luciferase vector and co-transfected with miR-4776 mimic into HBEpC cells. Transfection of miR-4776 mimic resulted in 80% reduction in luciferase activity compared to that from cells transfected with the negative control (Figure 8B). These results demonstrate that miR-4776 regulates NFKBIB mRNA expression through binding of the 3’ UTR.

Influenza viruses are a major concern due to the emergence in recent years of new and divergent strains that are potentially dangerous including H7N9 and the pandemic 2009 H1N1. There are reports that the virus also becomes drug resistant and the number of anti-viral drugs are limited. Therefore, understanding the host cell’s regulatory machinery used during influenza infection has the potential for identifying host cell targets in anti-influenza therapies. Regulation of NF-κB is a complex process, and its regulation involves a family of inhibitor proteins (NFKBIB), which form complexes with NF-κB, and prevents its translocation to the nucleus where activated NF-κB can affect the transcription of target genes. We also noted that the decreased expression of miR-4776 in bronchial epithelial cells was followed by an increase in NFKBIB mRNA and that subsequently resulted in an inactive NF-κB protein. IAV manipulates the NF-κB pathway for efficient replication of the virus and differentially regulates viral RNA synthesis [33]. Our data suggests that NF-κB is an important signaling molecule that plays a critical role in the replication of IAV and is modulated by the NFKBIB protein, which, in turn, is regulated by miR-4776.

## 4. Conclusions

In conclusion, the results presented here provide the first evidence that miR-4776 can modulate the expression of NFKBIB, the regulatory protein for a key transcription factor (NF-κB) that is necessary for cell proliferation, apoptosis and cell survival. Downregulation of NFKBIB mRNA by miR-4776 led to the production of active NF-κB that possibly modulates the increased viral survival. Identifying novel miRNAs for their target genes provides an alternative approach to reduce IAV infections and block influenza transmission. Validation of specific miRNAs as potential anti-viral agents will require further testing in animal models.

## Supplementary Materials

The following are available online at www.mdpi.com/1999-4915/9/5/94/s1, Table S1: Microarray data on miRNA expression on A549 cells exposed to IAV for 3 h. The log fold changes in expression of miRNAs that are up or down regulated on exposure to IAV are shown. AvgHy3-Average signal intensity, logFC-log fold change, Control-Mock; Treatment- Infected with H1N1.
