# Supplementary material for: Upregulation of miRNA-4776 in Influenza Virus Infected Bronchial Epithelial Cells Is Associated with Downregulation of NFKBIB and Increased Viral Survival
Source: Viruses. 2017 Apr 27;9(5):94. doi: 10.3390/v9050094 (PMC5454407; doi:10.3390/v9050094)
Supplement: Supplementary file 1 [file viruses-09-00094-s001.pdf]

Upregulation of miRNA-4776 in Influenza Virus Infected Bronchial Epithelial Cells Is Associated with Downregulation of NFKBIB and Increased Viral Survival

Sreekumar Othumpangat, Nicole B. Bryan, Donald H. Beezhold and John D. Noti

Table S1. IAV infection induced differential expression of miRNAs. Microarray analysis for miRNA expression was performed with RNA extracted from IAV infected A549 cells for 3 h. The log fold changes in expression of miRNAs that are up or down regulated on exposure to IAV are shown from the microarray. AvgHy3- Average signal intensity, logFC-log fold change, Control-Mock; Treatment-Infected with H1N1.

ProbeID	Annotation	AvgHy3	Control	Treatment	logFC
27537	ebv-miR-BART13	6.290	-1.016	-1.046	-0.030
17358	ebv-miR-BART16	5.503	0.119	0.167	0.048
42522	ebv-miR-BART19-3p	7.267	-0.551	-0.552	0.000
42613	ebv-miR-BART19-5p	5.382	0.607	0.714	0.107
17561	ebv-miR-BART6-3p	6.099	-1.549	-1.626	-0.076
17328	ebv-miR-BART8*	5.369	-0.493	-0.592	-0.099
42906	ebv-miR-BHRF1-1	5.975	-1.148	-1.116	0.032
42951	ebv-miR-BHRF1-2	6.768	-1.422	-1.556	-0.134
42782	hcmv-miR-UL148D	6.192	0.206	0.251	0.044
42530	hsa-let-7a-2-3p	5.299	-0.086	-0.130	-0.044
147162	hsa-let-7a-5p	7.746	0.463	0.164	-0.299
147165	hsa-let-7b-5p	7.835	0.297	0.400	0.103
145820	hsa-let-7c	6.308	0.409	0.318	-0.091
145633	hsa-let-7d-3p	5.325	-0.334	-0.351	-0.017
145968	hsa-let-7d-5p	6.053	0.354	0.051	-0.303
145846	hsa-let-7e-5p	8.699	-0.095	-0.288	-0.193
17752	hsa-let-7f-5p	5.855	0.684	0.448	-0.237
46438	hsa-let-7g-5p	7.333	0.617	0.247	-0.370
9938	hsa-let-7i-5p	8.809	0.383	0.172	-0.211
145943	hsa-miR-100-5p	6.106	0.493	0.304	-0.188
31026	hsa-miR-101-3p	5.774	0.581	0.917	0.335
10919	hsa-miR-103a-3p	8.281	0.510	0.437	-0.073
46801	hsa-miR-106a-5p	6.630	0.584	0.519	-0.065
17854	hsa-miR-106b-3p	6.081	0.465	0.587	0.122
19582	hsa-miR-106b-5p	7.258	0.533	0.833	0.300
10923	hsa-miR-107	6.835	0.344	0.228	-0.116
13485	hsa-miR-10a-5p	6.397	0.434	0.342	-0.093
10925	hsa-miR-10b-5p	5.233	0.431	0.318	-0.113
46258	hsa-miR-1184	6.594	-1.091	-1.177	-0.086
168595	hsa-miR-1185-2-3p	4.657	-1.907	-2.139	-0.232
46345	hsa-miR-1207-3p	5.889	1.283	1.401	0.118
46806	hsa-miR-1227	5.693	0.999	1.104	0.105
46531	hsa-miR-1231	5.207	0.744	0.719	-0.025
46624	hsa-miR-1236	5.943	0.398	0.530	0.132
46850	hsa-miR-1237	5.262	0.670	0.705	0.036
42898	hsa-miR-124-5p	5.362	0.387	0.379	-0.008
46404	hsa-miR-1244	5.426	0.605	0.753	0.148
168870	hsa-miR-1246	11.715	0.052	-0.151	-0.202

145977	hsa-miR-1247-5p	5.497	-0.126	-0.068	0.059
46427	hsa-miR-1248	5.890	0.534	0.358	-0.176
46210	hsa-miR-1249	6.619	1.811	1.915	0.104
46924	hsa-miR-1252	6.116	0.065	0.229	0.164
46380	hsa-miR-1255a	7.238	-0.040	-0.034	0.006
168811	hsa-miR-1255b-2-3p	5.569	-0.640	-0.643	-0.003
46869	hsa-miR-1258	5.177	0.259	0.257	-0.002
17942	hsa-miR-125a-3p	5.468	0.647	0.688	0.041
10928	hsa-miR-125a-5p	9.048	0.151	0.158	0.007
30787	hsa-miR-125b-5p	8.346	0.259	0.263	0.004
4610	hsa-miR-126-3p	5.462	0.553	0.430	-0.123
169412	hsa-miR-1260a	8.610	-1.142	-0.347	0.795
168619	hsa-miR-1260b	14.258	-0.912	-0.136	0.777
46732	hsa-miR-1264	7.169	0.341	0.836	0.494
46737	hsa-miR-1265	5.718	0.257	0.358	0.101
46623	hsa-miR-1273a	5.295	-0.836	-0.897	-0.061
148263	hsa-miR-1273e	5.497	-0.424	-0.461	-0.037
168846	hsa-miR-1273f	4.764	-1.113	-1.206	-0.094
168925	hsa-miR-1273g-3p	12.122	-0.049	-0.020	0.029
169082	hsa-miR-1275	8.065	-1.835	-1.794	0.041
45775	hsa-miR-1279	5.207	0.734	0.785	0.051
33902	hsa-miR-128	5.442	0.534	0.490	-0.044
168931	hsa-miR-1280	14.472	-0.964	-0.231	0.733
46634	hsa-miR-1281	6.246	1.205	1.304	0.098
46368	hsa-miR-1282	5.481	1.123	1.126	0.003
145981	hsa-miR-1285-3p	8.054	-0.292	-0.197	0.096
42571	hsa-miR-129-1-3p	7.004	0.083	0.004	-0.079
168568	hsa-miR-1290	5.218	-0.619	-0.802	-0.183
46788	hsa-miR-1299	5.989	0.119	0.179	0.060
46215	hsa-miR-1301	5.305	0.142	0.123	-0.019
46479	hsa-miR-1304-5p	5.826	-0.002	-0.052	-0.050
42681	hsa-miR-1307-3p	5.308	0.216	0.080	-0.136
42780	hsa-miR-1307-5p	4.825	-1.169	-1.260	-0.091
10138	hsa-miR-130a-3p	7.590	0.651	0.737	0.086
10936	hsa-miR-130b-3p	5.422	0.186	0.172	-0.014
46866	hsa-miR-1321	6.110	0.124	0.146	0.022
10943	hsa-miR-136-5p	5.182	0.483	0.595	0.112
148278	hsa-miR-138-2-3p	5.710	0.401	0.526	0.125
13140	hsa-miR-138-5p	7.860	0.490	0.477	-0.013
42630	hsa-miR-140-3p	5.738	0.137	0.034	-0.103
146072	hsa-miR-1469	8.215	-2.234	-2.171	0.064
146121	hsa-miR-1470	5.107	-0.013	0.121	0.135
10954	hsa-miR-147a	5.356	0.793	0.869	0.077
19585	hsa-miR-148b-3p	6.207	0.485	0.525	0.040
169087	hsa-miR-149-3p	6.270	-2.214	-2.325	-0.110
42810	hsa-miR-149-5p	5.494	0.260	0.146	-0.114
145678	hsa-miR-150-5p	6.469	-0.503	-0.628	-0.126
17463	hsa-miR-151a-3p	5.883	0.387	0.384	-0.003
11260	hsa-miR-151a-5p	6.423	0.617	0.542	-0.075
168871	hsa-miR-151a-5p/hsa-miR-151b	6.588	0.352	0.179	-0.173
17676	hsa-miR-152	5.540	0.616	0.587	-0.030
168672	hsa-miR-1587	6.257	-0.824	-0.795	0.029
27720	hsa-miR-15a-5p	6.926	0.794	1.019	0.225

17280	hsa-miR-15b-5p	8.661	0.355	0.294	-0.061
10967	hsa-miR-16-5p	8.564	0.825	0.785	-0.040
19588	hsa-miR-17-3p	5.141	0.290	0.322	0.031
169336	hsa-miR-17-5p	6.904	0.444	0.362	-0.082
42865	hsa-miR-181a-5p	6.855	0.164	0.090	-0.074
10972	hsa-miR-181b-5p	5.810	0.537	0.535	-0.002
169408	hsa-miR-181d	5.628	0.024	-0.131	-0.155
10975	hsa-miR-182-5p	5.858	0.396	0.209	-0.187
46266	hsa-miR-1825	5.561	0.972	1.060	0.088
46810	hsa-miR-1827	8.319	-0.577	-0.652	-0.075
17953	hsa-miR-183-3p	6.117	-1.005	-1.042	-0.037
10977	hsa-miR-183-5p	6.304	0.178	-0.072	-0.249
42902	hsa-miR-185-5p	5.716	0.213	0.143	-0.070
18739	hsa-miR-186-5p	5.172	0.185	0.210	0.025
145670	hsa-miR-18b-5p	5.529	0.448	0.556	0.107
148687	hsa-miR-1908	8.022	-1.764	-1.791	-0.027
146180	hsa-miR-1909-3p	5.077	-0.535	-0.580	-0.045
27536	hsa-miR-190a	5.222	0.644	0.620	-0.024
32731	hsa-miR-190b	5.292	0.590	0.718	0.128
10985	hsa-miR-191-5p	6.824	0.341	0.136	-0.205
146103	hsa-miR-1913	5.808	0.931	1.153	0.222
146091	hsa-miR-1914-5p	5.679	-0.689	-0.827	-0.139
146068	hsa-miR-1915-3p	5.204	-0.296	-0.362	-0.066
17732	hsa-miR-192-5p	6.766	0.598	0.644	0.046
10986	hsa-miR-193a-3p	5.500	0.101	0.190	0.089
46443	hsa-miR-193a-5p	5.294	0.398	0.385	-0.013
10987	hsa-miR-193b-3p	8.637	0.213	0.172	-0.041
10988	hsa-miR-194-5p	7.282	0.472	0.576	0.105
13148	hsa-miR-195-5p	5.101	0.029	-0.057	-0.086
42538	hsa-miR-196a-3p	5.731	-0.007	-0.191	-0.184
10990	hsa-miR-196a-5p	5.880	0.709	0.573	-0.136
42783	hsa-miR-197-3p	5.860	-0.145	-0.160	-0.015
146165	hsa-miR-1973	7.584	-0.285	-0.114	0.171
146140	hsa-miR-1976	5.764	0.324	0.449	0.125
10997	hsa-miR-19a-3p	6.378	0.634	0.909	0.275
10998	hsa-miR-19b-3p	6.333	0.537	0.869	0.332
147186	hsa-miR-200b-3p	5.083	0.300	0.275	-0.025
42502	hsa-miR-204-3p	8.541	-2.157	-1.972	0.185
169411	hsa-miR-205-3p	5.844	-0.519	-0.492	0.027
46917	hsa-miR-205-5p	5.279	0.643	0.758	0.115
145845	hsa-miR-20a-5p	7.751	0.758	0.769	0.011
17882	hsa-miR-20b-3p	5.908	0.050	0.091	0.041
42640	hsa-miR-20b-5p	5.863	0.586	0.575	-0.011
42524	hsa-miR-21-3p	6.421	0.090	0.250	0.160
147506	hsa-miR-21-5p	11.134	0.861	1.203	0.342
145852	hsa-miR-210	5.604	0.374	-0.060	-0.434
146161	hsa-miR-2115-3p	6.404	-0.051	-0.125	-0.074
146010	hsa-miR-2116-5p	7.001	-0.083	-0.094	-0.011
11014	hsa-miR-214-3p	5.578	-0.858	-0.813	0.045
11020	hsa-miR-22-3p	9.123	0.509	0.561	0.052
42532	hsa-miR-22-5p	5.621	0.505	0.528	0.022
11022	hsa-miR-221-3p	7.490	0.334	0.278	-0.056
11023	hsa-miR-222-3p	8.548	0.271	0.274	0.003
11024	hsa-miR-223-3p	5.133	0.017	0.095	0.079

146163	hsa-miR-224-3p	5.321	0.041	0.039	-0.002
168772	hsa-miR-224-5p	5.609	0.051	-0.130	-0.181
148247	hsa-miR-2355-3p	6.468	0.145	0.110	-0.035
42744	hsa-miR-23a-3p	9.498	0.438	0.626	0.187
42638	hsa-miR-23a-5p	5.420	0.591	0.658	0.066
169330	hsa-miR-23b-3p	9.841	0.447	0.556	0.109
148217	hsa-miR-23c	7.035	0.181	0.233	0.051
17506	hsa-miR-24-3p	10.415	0.530	0.629	0.099
42682	hsa-miR-25-3p	6.355	0.585	0.387	-0.199
42929	hsa-miR-25-5p	7.210	-1.244	-1.096	0.148
168958	hsa-miR-2681-5p	6.098	-0.350	-0.280	0.070
147198	hsa-miR-26a-5p	5.152	0.088	-0.100	-0.188
146008	hsa-miR-26b-5p	6.744	0.488	0.293	-0.195
46483	hsa-miR-27a-3p	7.104	0.404	0.628	0.223
147199	hsa-miR-27b-3p	8.913	0.674	0.797	0.124
42823	hsa-miR-27b-5p	5.553	0.385	0.321	-0.064
46264	hsa-miR-298	5.811	0.154	0.493	0.338
11038	hsa-miR-299-5p	5.168	0.285	0.325	0.040
168687	hsa-miR-29a-3p	8.939	0.230	0.375	0.145
145638	hsa-miR-29a-5p	5.573	0.620	0.750	0.130
17810	hsa-miR-29b-1-5p	6.111	0.901	0.819	-0.083
11040	hsa-miR-29b-3p	7.759	0.408	0.894	0.486
11041	hsa-miR-29c-3p	5.985	0.278	0.439	0.162
13143	hsa-miR-301a-3p	5.736	0.640	0.939	0.300
147203	hsa-miR-302a-3p	7.577	-0.719	-0.777	-0.058
11044	hsa-miR-302c-3p	5.305	0.873	0.895	0.022
11045	hsa-miR-302c-5p	5.512	0.217	0.318	0.101
146086	hsa-miR-30a-5p	6.472	0.269	0.274	0.005
146112	hsa-miR-30b-5p	6.567	0.337	0.364	0.027
42923	hsa-miR-30c-5p	7.086	0.230	0.302	0.072
19596	hsa-miR-30d-5p	6.511	0.460	0.517	0.057
145676	hsa-miR-30e-3p	5.782	0.451	0.321	-0.130
28191	hsa-miR-30e-5p	5.859	0.473	0.577	0.104
46320	hsa-miR-31-3p	5.846	0.365	0.529	0.164
11052	hsa-miR-31-5p	8.567	0.331	0.251	-0.081
169380	hsa-miR-3124-3p	10.084	-1.363	-1.265	0.098
168698	hsa-miR-3127-3p	5.198	0.523	0.599	0.076
147820	hsa-miR-3133	8.051	-0.084	0.016	0.100
147988	hsa-miR-3135a	5.136	-0.421	-0.473	-0.052
169319	hsa-miR-3136-3p	7.613	-0.396	-0.308	0.088
147923	hsa-miR-3142	6.007	-0.208	-0.074	0.134
147904	hsa-miR-3148	5.714	-0.079	-0.130	-0.051
147806	hsa-miR-3149	6.992	-0.052	0.017	0.069
169232	hsa-miR-3156-3p	9.501	-0.458	-0.135	0.323
168653	hsa-miR-3158-5p	8.318	-3.885	-3.878	0.007
147739	hsa-miR-3161	5.510	-0.515	-0.490	0.024
147891	hsa-miR-3175	7.835	-0.184	-0.120	0.064
147595	hsa-miR-3178	8.912	-3.022	-2.915	0.107
147667	hsa-miR-3182	10.544	0.487	0.901	0.414
147805	hsa-miR-3183	5.505	-0.344	-0.244	0.100
148000	hsa-miR-3195	6.255	-1.427	-1.521	-0.094
147817	hsa-miR-3196	6.990	-2.144	-2.135	0.009
29575	hsa-miR-32-3p	7.652	-0.049	0.032	0.081
11053	hsa-miR-32-5p	5.272	0.299	0.671	0.371

147851	hsa-miR-3201	5.215	-0.635	-0.702	-0.067
146158	hsa-miR-3202	6.365	-1.148	-1.099	0.048
27533	hsa-miR-320a	7.196	0.156	0.155	-0.001
46324	hsa-miR-320b	7.198	0.122	0.130	0.008
169363	hsa-miR-320c	6.552	-0.077	-0.094	-0.017
145708	hsa-miR-324-3p	5.403	0.321	0.318	-0.003
42887	hsa-miR-331-3p	7.584	0.081	0.285	0.205
145745	hsa-miR-335-3p	8.464	-0.407	-0.270	0.137
11065	hsa-miR-335-5p	4.974	0.126	0.149	0.022
42739	hsa-miR-339-5p	5.549	0.098	-0.035	-0.132
145859	hsa-miR-33a-5p	4.868	0.019	0.371	0.352
29872	hsa-miR-340-5p	5.752	0.029	0.245	0.216
32884	hsa-miR-342-3p	5.048	-0.028	-0.101	-0.073
168586	hsa-miR-34a-5p	8.221	0.257	0.098	-0.159
11073	hsa-miR-34b-5p	5.091	0.407	0.396	-0.011
11074	hsa-miR-34c-5p	5.327	0.382	0.404	0.022
168994	hsa-miR-3591-5p	6.128	-0.191	-0.182	0.009
148335	hsa-miR-3606	5.227	-0.191	-0.055	0.136
148420	hsa-miR-3607-3p	8.284	0.443	0.385	-0.058
148418	hsa-miR-3607-5p	7.355	0.453	0.343	-0.110
168689	hsa-miR-361-3p	5.190	-0.253	-0.301	-0.048
14301	hsa-miR-361-5p	6.427	0.482	0.409	-0.073
148465	hsa-miR-3611	6.789	-0.503	-0.513	-0.010
148493	hsa-miR-3613-3p	12.945	0.925	1.010	0.085
148413	hsa-miR-3614-3p	5.924	1.506	1.649	0.143
148317	hsa-miR-3621	6.846	-1.573	-1.618	-0.045
148132	hsa-miR-3622a-3p	5.594	0.200	0.252	0.052
27544	hsa-miR-363-5p	6.417	0.484	0.404	-0.080
148481	hsa-miR-3646	8.748	-0.796	-0.416	0.380
148327	hsa-miR-3651	7.777	0.024	0.109	0.085
148377	hsa-miR-3653	6.505	-0.099	-0.087	0.012
148379	hsa-miR-3654	7.168	0.028	0.045	0.018
148228	hsa-miR-3656	6.051	-0.813	-0.696	0.117
11078	hsa-miR-365a-3p/hsa-miR-365b-3p	7.484	0.295	0.093	-0.202
148206	hsa-miR-3664-5p	5.739	1.253	1.424	0.171
148234	hsa-miR-3667-5p	8.837	-0.265	-0.285	-0.020
148214	hsa-miR-3675-3p	6.170	0.099	0.242	0.143
148410	hsa-miR-3676-3p	7.962	-0.872	-0.877	-0.005
168763	hsa-miR-3676-5p	7.308	-0.085	0.154	0.239
148038	hsa-miR-3679-3p	7.443	1.505	1.720	0.215
148599	hsa-miR-3680-5p	7.306	0.216	0.479	0.263
148032	hsa-miR-3685	9.186	0.125	0.454	0.329
148156	hsa-miR-3686	9.770	-0.459	-0.306	0.153
148085	hsa-miR-3687	7.661	-1.619	-1.415	0.204
148077	hsa-miR-3690	5.700	-0.353	-0.401	-0.048
148282	hsa-miR-3714	5.177	0.547	0.657	0.110
168978	hsa-miR-371b-5p	10.350	-2.333	-2.431	-0.097
27545	hsa-miR-373-5p	5.244	-0.488	-0.642	-0.153
145844	hsa-miR-374a-5p	5.810	0.418	0.656	0.238
148098	hsa-miR-374b-5p	6.004	0.461	0.538	0.077
148430	hsa-miR-374c-5p	6.010	0.370	0.386	0.016
148668	hsa-miR-378a-3p	6.206	0.241	0.216	-0.025
147755	hsa-miR-378c	5.495	0.381	0.370	-0.011

169266	hsa-miR-378d	5.144	-0.183	-0.268	-0.085
148361	hsa-miR-3911	5.161	0.213	0.140	-0.072
148495	hsa-miR-3915	6.389	-0.449	-0.500	-0.051
148154	hsa-miR-3922-3p	5.225	0.847	0.955	0.108
148049	hsa-miR-3924	5.921	0.446	0.538	0.092
148265	hsa-miR-3935	6.570	-0.551	-0.598	-0.048
148349	hsa-miR-3938	5.861	0.177	0.181	0.004
168637	hsa-miR-3940-5p	10.413	-1.950	-2.079	-0.129
148285	hsa-miR-3941	7.269	-0.194	-0.199	-0.006
148577	hsa-miR-3943	5.171	0.310	0.381	0.072
169024	hsa-miR-3960	11.369	-2.249	-2.307	-0.058
169316	hsa-miR-3976	8.952	-0.940	-0.876	0.065
42730	hsa-miR-423-3p	7.841	-0.036	0.039	0.076
27565	hsa-miR-423-5p	7.793	-0.572	-0.663	-0.091
42965	hsa-miR-424-5p	7.068	0.476	0.795	0.319
17608	hsa-miR-425-5p	5.838	0.377	0.468	0.091
147631	hsa-miR-4258	5.986	0.487	0.516	0.029
147942	hsa-miR-4268	8.725	-1.290	-1.273	0.017
147751	hsa-miR-4274	6.123	1.471	1.583	0.111
147767	hsa-miR-4279	9.225	-1.522	-1.363	0.159
169129	hsa-miR-4284	14.998	0.167	0.029	-0.139
147604	hsa-miR-4285	8.488	-2.204	-2.255	-0.052
169409	hsa-miR-4286	7.406	-0.360	0.062	0.422
147588	hsa-miR-4288	8.243	0.378	0.340	-0.038
147735	hsa-miR-4289	5.691	0.157	-0.007	-0.163
169282	hsa-miR-4290	9.314	-1.462	-1.377	0.085
147616	hsa-miR-4291	7.164	0.185	0.169	-0.016
147632	hsa-miR-4297	6.877	0.294	0.407	0.113
147614	hsa-miR-4299	6.737	-0.841	-0.935	-0.093
169407	hsa-miR-4301	10.692	0.032	0.139	0.107
147722	hsa-miR-4306	7.445	-0.956	-0.993	-0.036
145705	hsa-miR-431-5p	5.235	0.734	0.699	-0.035
147907	hsa-miR-4312	5.793	0.691	0.779	0.088
147776	hsa-miR-4317	5.621	0.228	0.252	0.024
147671	hsa-miR-4323	5.209	-0.344	-0.281	0.063
168980	hsa-miR-4324	6.033	-0.373	-0.340	0.033
147926	hsa-miR-4329	5.465	-0.277	-0.337	-0.060
169358	hsa-miR-4417	5.854	-1.037	-0.568	0.469
169272	hsa-miR-4419b	9.742	-1.226	-1.068	0.158
169381	hsa-miR-4421	7.948	-0.685	-0.706	-0.022
168768	hsa-miR-4423-5p	6.352	-2.175	-2.013	0.162
168709	hsa-miR-4429	6.626	-0.279	-0.250	0.029
168928	hsa-miR-4431	4.818	-1.873	-1.768	0.104
169260	hsa-miR-4436b-3p	4.977	-0.944	-0.962	-0.018
169171	hsa-miR-4436b-5p	5.772	-0.248	-0.229	0.019
169188	hsa-miR-4443	11.709	-1.137	-0.508	0.629
168971	hsa-miR-4449	5.198	-1.494	-1.553	-0.059
169167	hsa-miR-4451	6.064	-0.895	-0.850	0.045
169015	hsa-miR-4454	14.685	0.397	0.765	0.368
169305	hsa-miR-4455	7.794	-0.113	-0.023	0.090
168919	hsa-miR-4456	12.782	-0.074	0.297	0.371
169143	hsa-miR-4459	5.942	-1.192	-1.247	-0.055
168814	hsa-miR-4463	5.334	-1.227	-1.376	-0.149
169285	hsa-miR-4467	9.966	-1.884	-1.837	0.048

169320	hsa-miR-4468	6.282	-0.545	-0.651	-0.106
169170	hsa-miR-4472	5.169	-0.571	-0.565	0.006
168904	hsa-miR-4473	5.932	-0.831	-0.925	-0.095
168640	hsa-miR-4475	8.759	-0.958	-0.989	-0.030
168805	hsa-miR-4478	6.192	-0.795	-0.735	0.061
169395	hsa-miR-4484	6.740	-0.768	-0.613	0.156
46808	hsa-miR-4485	4.969	-1.311	-1.235	0.076
168815	hsa-miR-4488	4.787	-1.471	-1.715	-0.245
169110	hsa-miR-4497	9.550	-2.163	-2.292	-0.129
169385	hsa-miR-4500	8.135	0.233	0.165	-0.068
168893	hsa-miR-4505	7.064	-1.662	-1.619	0.043
168572	hsa-miR-4507	4.982	-0.780	-0.784	-0.004
168998	hsa-miR-4508	6.040	-0.887	-1.159	-0.272
168917	hsa-miR-4511	4.861	-1.899	-1.698	0.202
169194	hsa-miR-4513	4.533	-2.104	-2.271	-0.167
168948	hsa-miR-4514	5.363	-1.545	-1.310	0.235
168802	hsa-miR-4516	6.950	-1.739	-2.032	-0.293
169199	hsa-miR-4518	5.209	-0.390	-0.396	-0.006
169326	hsa-miR-451b	5.930	0.019	0.036	0.017
29379	hsa-miR-452-5p	5.952	0.326	0.317	-0.009
169159	hsa-miR-4521	6.034	-0.228	-0.974	-0.746
168638	hsa-miR-4530	7.649	-1.693	-1.852	-0.160
168661	hsa-miR-4531	7.793	-1.099	-0.998	0.101
168844	hsa-miR-4532	7.264	-1.212	-1.097	0.115
168639	hsa-miR-4533	7.199	-0.348	-0.452	-0.104
148620	hsa-miR-454-3p	5.118	0.361	0.246	-0.115
168702	hsa-miR-4540	6.212	-0.767	-0.654	0.113
28950	hsa-miR-455-3p	5.362	0.173	0.113	-0.059
13179	hsa-miR-455-5p	5.108	0.466	0.512	0.046
169102	hsa-miR-4639-3p	10.608	0.050	0.310	0.260
169183	hsa-miR-4644	7.974	-1.174	-1.272	-0.098
168750	hsa-miR-4645-5p	5.633	-0.384	-0.291	0.093
168950	hsa-miR-4646-3p	5.426	-0.436	-0.453	-0.017
168605	hsa-miR-4653-3p	5.284	-1.439	-1.357	0.082
169238	hsa-miR-4654	4.867	-1.578	-1.718	-0.139
46731	hsa-miR-4657	8.700	-0.226	-0.076	0.150
169035	hsa-miR-4658	4.973	-0.964	-0.966	-0.003
169079	hsa-miR-4667-5p	6.356	-0.623	-0.585	0.037
168798	hsa-miR-4668-5p	13.560	0.807	0.903	0.096
168832	hsa-miR-4674	5.368	-1.041	-1.413	-0.372
168935	hsa-miR-4687-3p	5.708	-0.982	-0.842	0.140
168648	hsa-miR-4687-5p	5.798	-0.515	-0.405	0.110
169253	hsa-miR-4690-5p	4.965	-1.528	-1.657	-0.129
168670	hsa-miR-4694-5p	4.782	-1.274	-1.166	0.108
169070	hsa-miR-4695-3p	8.733	-0.968	-0.267	0.701
169228	hsa-miR-4698	5.567	-0.873	-1.011	-0.138
169081	hsa-miR-4707-3p	5.934	-1.102	-1.083	0.018
168944	hsa-miR-4707-5p	5.910	-1.351	-1.513	-0.162
169028	hsa-miR-4708-3p	11.046	-2.175	-2.176	-0.001
169204	hsa-miR-4709-3p	8.007	-0.753	-0.850	-0.096
169023	hsa-miR-4712-3p	6.461	-0.534	-0.486	0.048
169311	hsa-miR-4714-5p	9.575	-0.098	0.102	0.200
168674	hsa-miR-4716-5p	5.494	-0.097	0.028	0.125
169323	hsa-miR-4723-3p	6.612	1.109	1.291	0.182

169295	hsa-miR-4725-3p	4.945	-1.644	-1.786	-0.142
169193	hsa-miR-4725-5p	6.124	-0.649	-0.649	0.000
169031	hsa-miR-4726-5p	7.366	-0.880	-0.923	-0.042
169182	hsa-miR-4728-3p	7.914	-1.194	-1.097	0.097
168668	hsa-miR-4732-3p	6.277	-0.590	-0.476	0.113
169239	hsa-miR-4732-5p	5.403	-0.470	-0.429	0.041
169265	hsa-miR-4733-3p	5.761	-1.572	-1.821	-0.249
168696	hsa-miR-4739	5.350	-1.319	-1.413	-0.094
168722	hsa-miR-4742-3p	6.767	-1.731	-1.728	0.003
169393	hsa-miR-4747-5p	7.894	-1.335	-1.258	0.077
169399	hsa-miR-4750	7.100	-1.581	-1.644	-0.063
168955	hsa-miR-4756-3p	5.437	-0.548	-0.615	-0.067
169100	hsa-miR-4758-3p	5.986	-0.418	-0.467	-0.049
169264	hsa-miR-4762-5p	5.392	-0.339	-0.447	-0.108
169130	hsa-miR-4764-3p	9.983	-0.312	-0.211	0.101
168943	hsa-miR-4769-3p	6.110	0.386	0.521	0.135
168781	hsa-miR-4776-3p	5.145	-0.928	-1.208	-0.280
168557	hsa-miR-4777-5p	5.220	-0.943	-0.990	-0.047
168915	hsa-miR-4780	9.894	0.269	0.216	-0.053
169271	hsa-miR-4784	5.691	-0.638	-0.715	-0.077
169120	hsa-miR-4787-3p	5.571	-1.157	-1.100	0.057
169050	hsa-miR-4787-5p	11.123	-2.338	-2.322	0.016
169116	hsa-miR-4788	6.093	-2.946	-2.915	0.031
168995	hsa-miR-4791	9.059	0.163	0.033	-0.130
168776	hsa-miR-4795-3p	6.514	-1.324	-1.251	0.073
169189	hsa-miR-4795-5p	6.869	-0.501	-0.514	-0.013
169022	hsa-miR-4797-5p	5.256	-0.766	-0.364	0.402
169313	hsa-miR-4800-3p	9.939	-0.806	-0.820	-0.013
169390	hsa-miR-4800-5p	4.815	-1.294	-1.394	-0.100
169096	hsa-miR-4804-3p	4.799	-1.264	-1.129	0.135
148682	hsa-miR-483-3p	8.891	-1.789	-1.656	0.134
42654	hsa-miR-483-5p	5.682	-0.370	-0.406	-0.036
145753	hsa-miR-484	5.580	0.370	0.454	0.084
42694	hsa-miR-485-3p	5.723	-0.464	-0.554	-0.091
32946	hsa-miR-486-5p	5.248	0.447	0.531	0.084
14285	hsa-miR-487b	5.375	-0.116	0.151	0.268
42703	hsa-miR-490-3p	5.105	-0.851	-0.843	0.008
17822	hsa-miR-490-5p	5.283	-0.104	-0.153	-0.049
147701	hsa-miR-491-3p	8.723	-0.297	-0.165	0.131
42661	hsa-miR-492	5.722	-0.829	-1.063	-0.234
148059	hsa-miR-493-5p	6.919	0.053	0.181	0.128
42442	hsa-miR-498	6.120	-0.375	-0.429	-0.053
169145	hsa-miR-5000-3p	5.753	-0.405	-0.353	0.052
168656	hsa-miR-5002-3p	6.020	-0.692	-0.645	0.047
168769	hsa-miR-5002-5p	7.119	-0.796	-0.862	-0.067
169296	hsa-miR-5004-5p	5.141	-2.361	-2.381	-0.020
169136	hsa-miR-5006-3p	5.687	-0.642	-0.649	-0.007
17875	hsa-miR-500a-5p	5.238	0.441	0.486	0.045
145675	hsa-miR-501-5p	5.574	0.221	0.209	-0.012
42490	hsa-miR-505-5p	5.578	0.266	0.167	-0.099
11139	hsa-miR-507	5.325	0.839	0.950	0.111
168887	hsa-miR-5089	5.032	-0.500	-0.403	0.096
168878	hsa-miR-5100	13.423	0.324	0.607	0.283
42581	hsa-miR-513a-5p	8.479	-0.704	-0.675	0.029

46789	hsa-miR-513b	5.249	0.104	0.141	0.037
11151	hsa-miR-516b-5p	5.459	-0.362	-0.383	-0.021
169185	hsa-miR-5187-3p	6.296	-1.268	-1.382	-0.115
148641	hsa-miR-518b	5.252	-0.179	-0.161	0.018
13137	hsa-miR-518e-5p/hsa-miR-519a-5p/hsa-miR-519b-5p/hsa-miR-519c-5p/hsa-miR-522-5p/hsa-miR-523-5p	5.508	-0.359	-0.476	-0.118
168717	hsa-miR-5193	7.619	-0.130	0.004	0.134
169274	hsa-miR-5196-3p	5.441	-1.239	-1.262	-0.023
46221	hsa-miR-519d	5.278	0.702	0.734	0.032
13132	hsa-miR-519e-5p	5.614	-0.924	-0.923	0.001
14272	hsa-miR-542-3p	5.279	0.285	0.339	0.054
169312	hsa-miR-548an	6.140	-0.950	-1.219	-0.270
169009	hsa-miR-548ap-5p/hsa-miR-548j	5.786	-0.777	-0.740	0.037
168951	hsa-miR-548as-3p	9.910	0.025	0.220	0.195
145789	hsa-miR-550a-3-5p/hsa-miR-550a-5p	5.277	0.068	0.088	0.021
148288	hsa-miR-550b-3p	5.267	-0.072	-0.205	-0.133
17272	hsa-miR-551a	5.683	-0.603	-0.622	-0.018
17668	hsa-miR-552	5.735	-1.179	-1.197	-0.018
168933	hsa-miR-5581-3p	6.877	-0.624	-0.333	0.291
168922	hsa-miR-5584-3p	5.907	-0.375	-0.471	-0.096
168868	hsa-miR-5681b	9.757	-0.789	-0.608	0.181
169169	hsa-miR-5684	11.027	-0.259	-0.176	0.084
169376	hsa-miR-5701	10.257	0.151	0.294	0.143
169211	hsa-miR-5704	9.711	-0.314	0.060	0.374
28966	hsa-miR-574-3p	6.368	-0.091	-0.150	-0.059
27740	hsa-miR-574-5p	7.211	0.107	0.122	0.014
42446	hsa-miR-576-5p	5.223	0.371	0.403	0.032
42800	hsa-miR-582-5p	5.268	0.161	0.231	0.070
145647	hsa-miR-584-5p	5.830	-0.300	-0.464	-0.164
17546	hsa-miR-585	5.428	0.071	0.023	-0.049
17503	hsa-miR-590-5p	4.914	0.222	0.348	0.127
42504	hsa-miR-593-3p	5.164	0.669	0.681	0.012
17377	hsa-miR-600	5.995	0.216	0.049	-0.167
17498	hsa-miR-601	5.316	0.466	0.515	0.049
27551	hsa-miR-612	5.181	-0.356	-0.371	-0.015
27672	hsa-miR-615-3p	5.148	-0.062	-0.037	0.024
17336	hsa-miR-618	5.570	1.161	1.198	0.037
148652	hsa-miR-620	5.266	-0.207	-0.278	-0.071
17493	hsa-miR-622	5.303	-0.335	-0.309	0.026
17566	hsa-miR-629-3p	5.140	-0.791	-0.409	0.382
17961	hsa-miR-629-5p	5.168	0.405	0.383	-0.022
17327	hsa-miR-630	5.425	-0.238	-0.272	-0.034
42591	hsa-miR-634	9.146	-1.257	-1.389	-0.133
42750	hsa-miR-636	5.659	0.274	0.250	-0.024
42832	hsa-miR-638	5.934	-0.175	-0.161	0.014
168642	hsa-miR-642b-3p	6.433	-2.528	-2.587	-0.059
169034	hsa-miR-642b-5p	9.787	-1.618	-1.545	0.073
168882	hsa-miR-644b-3p	9.619	-0.945	-0.775	0.170
168963	hsa-miR-644b-5p	7.276	0.290	0.364	0.074

42827	hsa-miR-652-3p	5.558	0.274	0.102	-0.172
145933	hsa-miR-652-5p	5.350	-0.524	-0.505	0.019
42843	hsa-miR-654-5p	5.084	-0.775	-0.911	-0.137
42749	hsa-miR-659-3p	5.407	-0.120	-0.124	-0.004
169375	hsa-miR-660-3p	10.046	-1.086	-1.079	0.007
169388	hsa-miR-663a	6.408	-1.798	-1.818	-0.019
145976	hsa-miR-663b	5.263	-0.757	-0.759	-0.002
145973	hsa-miR-664-3p	6.747	-0.250	-0.217	0.034
46829	hsa-miR-664-5p	5.517	0.486	0.499	0.013
145768	hsa-miR-665	7.026	-1.603	-1.449	0.154
145701	hsa-miR-668	6.427	0.257	0.174	-0.083
42859	hsa-miR-675-3p	5.741	0.977	1.051	0.074
42761	hsa-miR-675-5p	5.679	-0.411	-0.466	-0.054
29490	hsa-miR-7-5p	6.411	0.708	0.751	0.043
146196	hsa-miR-711	5.556	-1.107	-1.070	0.037
146064	hsa-miR-718	5.744	0.750	0.890	0.140
168838	hsa-miR-720	11.045	-0.084	0.435	0.520
27568	hsa-miR-744-5p	7.828	-0.453	-0.336	0.117
146111	hsa-miR-767-5p	7.742	0.158	0.209	0.051
42808	hsa-miR-874	7.389	-2.075	-2.110	-0.035
148622	hsa-miR-877-3p	6.845	-0.046	-0.057	-0.011
30033	hsa-miR-877-5p	5.709	0.219	0.247	0.028
46259	hsa-miR-885-5p	5.963	1.250	1.340	0.090
27961	hsa-miR-891a	5.306	-0.239	-0.287	-0.048
148621	hsa-miR-892a	5.383	0.620	0.701	0.081
4040	hsa-miR-9-5p	5.378	0.718	0.666	-0.052
42801	hsa-miR-92a-2-5p	5.209	0.672	0.670	-0.002
145693	hsa-miR-92a-3p	7.774	0.238	0.161	-0.077
145897	hsa-miR-92b-3p	5.687	-0.344	-0.352	-0.008
30687	hsa-miR-93-5p	7.990	0.384	0.256	-0.129
17863	hsa-miR-934	5.114	-0.265	-0.352	-0.087
145742	hsa-miR-935	6.264	0.125	-0.069	-0.194
42696	hsa-miR-943	5.692	0.487	0.463	-0.024
13147	hsa-miR-96-5p	5.814	0.767	0.889	0.123
11182	hsa-miR-98	6.335	0.596	0.485	-0.111
17898	hsa-miR-99b-3p	5.527	0.373	0.279	-0.094
11184	hsa-miR-99b-5p	7.349	0.140	0.043	-0.097
28302	hsa-miRPlus-A1015	8.942	-0.074	0.130	0.204
42793	hsa-miRPlus-A1072	5.852	-0.736	-0.524	0.212
17858	hsa-miRPlus-A1073	5.860	1.081	1.196	0.115
169416	hsa-miRPlus-A1086	6.567	-0.183	-0.076	0.107
17848	hsa-miRPlus-A1087	5.986	0.250	0.440	0.190
42838	hsa-miRPlus-C1076	5.190	-0.321	-0.366	-0.045
146113	hsa-miRPlus-G1246-3p	7.497	0.396	0.324	-0.072
147975	hsa-miRPlus-J1003	5.367	0.278	0.298	0.020
147415	hsa-miRPlus-J1011	5.296	-0.479	-0.483	-0.003
147576	hsv1-miR-H1*	5.880	1.045	1.186	0.140
147889	hsv1-miR-H14-3p	5.444	-0.151	-0.269	-0.118
147804	hsv1-miR-H17	6.058	-1.302	-1.314	-0.012
147830	hsv1-miR-H18	5.159	-0.915	-1.119	-0.204
147718	hsv1-miR-H3*	5.127	-0.274	-0.285	-0.012
146159	hsv1-miR-H4-3p	8.083	-0.031	-0.008	0.022
146098	hsv1-miR-H5-3p	5.584	-0.237	-0.197	0.040
146117	hsv1-miR-H6-3p	8.708	-0.573	-0.665	-0.091

146090	hsv1-miR-H7*	6.825	-0.766	-0.913	-0.147
146042	hsv1-miR-H8*	6.382	0.644	0.683	0.038
147803	hsv2-miR-H10	6.118	-1.027	-1.074	-0.047
147864	hsv2-miR-H24	5.505	0.267	0.398	0.131
147884	hsv2-miR-H25	6.122	-1.372	-1.410	-0.039
147900	hsv2-miR-H6*	7.158	-0.191	-0.181	0.010
147790	hsv2-miR-H7-3p	5.918	-0.640	-0.796	-0.156
147840	hsv2-miR-H9-3p	8.160	-1.833	-2.042	-0.210
42656	kshv-miR-K12-10a	6.170	1.448	1.615	0.167
42624	kshv-miR-K12-10b	5.974	1.373	1.445	0.072
42795	kshv-miR-K12-3	6.459	-1.344	-1.423	-0.079
147885	kshv-miR-K12-5*	7.240	-0.259	-0.004	0.255
17488	kshv-miR-K12-6-3p	6.581	-1.351	-1.325	0.026
147936	kshv-miR-K12-8*	5.223	-0.318	-0.228	0.090
146169	mcv-miR-M1-3p	6.344	-2.433	-2.216	0.216
19011	SNORD10	10.201	-0.040	0.082	0.122
145666	SNORD110	8.120	0.216	0.228	0.013
19005	SNORD118	8.248	0.133	0.059	-0.074
19606	SNORD12	6.634	0.027	-0.022	-0.050
19603	SNORD13	10.462	-0.010	0.083	0.092
19013	SNORD14B	8.887	-0.780	-0.914	-0.134
19008	SNORD2	9.942	0.409	0.390	-0.019
19007	SNORD3@	11.910	0.003	-0.131	-0.134
46204	SNORD38B	9.345	0.287	0.372	0.084
46206	SNORD44	10.311	0.106	0.054	-0.052
46205	SNORD48	9.361	0.176	0.198	0.021
46203	SNORD49A	8.982	0.337	0.192	-0.145
19604	SNORD4A	9.169	0.120	0.224	0.104
19605	SNORD6	10.463	0.077	0.285	0.209
145661	SNORD65	6.668	0.114	0.163	0.049
145663	SNORD68	10.671	-0.097	-0.129	-0.032
17492	sv40-miR-S1-5p	8.239	-1.649	-1.708	-0.058

© 2017 by the authors. Licensee MDPI, Basel, Switzerland. This article is an open access article distributed under the terms and conditions of the Creative Commons Attribution (CC BY) license (http://creativecommons.org/licenses/by/4.0/).
